# Differential expression of polyamine biosynthetic pathways in skin lesions and in plasma reveals distinct profiles in diffuse cutaneous leishmaniasis

**DOI:** 10.1038/s41598-020-67432-5

**Published:** 2020-06-29

**Authors:** Hayna Malta-Santos, Jaqueline França-Costa, Amanda Macedo, Artur T. L. Queiroz, Kiyoshi F. Fukutani, Sandra Marcia Muxel, Ricardo Khouri, Johan Van Weyenbergh, Viviane Boaventura, Aldina Barral, Jackson M. Costa, Eny Iochevet Segal Floh, Bruno B. Andrade, Lucile M. Floeter-Winter, Valéria M. Borges

**Affiliations:** 1 Universidade Federal da Bahia, Salvador, Brazil; 2 Instituto Gonçalo Moniz (IGM), Fundação Oswaldo Cruz (FIOCRUZ), Salvador, Brazil; 3 Departamento de Botânica, Instituto de Ciências Biomédicas, Universidade de São Paulo, São Paulo, Brazil; 4 Multinational Organization Network Sponsoring Translational and Epidemiological Research (MONSTER) Initiative, Salvador, Brazil; 5 Departamento de Parasitologia, Instituto de Ciências Biomédicas, Universidade de São Paulo, São Paulo, Brazil; 6 Department of Microbiology and Immunology, Rega Institute for Medical Research, University of Leuven, Leuven, Belgium; 7 Universidade Federal do Maranhão, São Luis, Brazil; 8 Escola Bahiana de Medicina e Saúde Pública, Salvador, Brazil; 9 Universidade Salvador (UNIFACS), Laureate Universities, Salvador, Brazil

**Keywords:** Parasitic infection, Cell biology

## Abstract

Tegumentary leishmaniasis (TL) is a parasitic disease that can result in wide spectrum clinical manifestations. It is necessary to understand host and parasite determinants of clinical outcomes to identify novel therapeutic targets. Previous studies have indicated that the polyamine biosynthetic pathway is critical for *Leishmania* growth and survival. Despite its importance, expression of the such pathway has not been previously investigated in TL patients. We performed an exploratory analysis employing Systems Biology tools to compare circulating polyamines and amino acid concentration as well as polyamine pathway gene expression in cutaneous lesions patients presenting with distinct TL disease presentations. Diffuse cutaneous leishmaniasis (DCL) was associated with higher concentrations of amino acids, polyamines and its substrate transporters than mucosal cutaneous leishmaniasis or localized cutaneous leishmaniasis. In addition, the RNA expression of polyamine-related genes of patients lesions from two separate cohorts demonstrated that differential activation of this pathway is associated with parasite loads and able to discriminate the clinical spectrum of TL. Taken together, our findings highlight a new aspect of DCL immunopathogenesis indicating that the polyamine pathway may be explored as a novel therapeutic target to control disease burden.

## Introduction

*Leishmania* infection causes Tegumentary Leishmaniasis (TL), which exhibits a broad spectrum of clinical manifestations. Clinical forms vary from self-healing localized cutaneous leishmaniasis (LCL), with a moderate cell-mediated immune response, to more severe forms such as the hyper-inflammatory mucocutaneous leishmaniasis (MCL); both conditions are caused by *L. braziliensis*. A less common disease manifestation is the diffuse cutaneous leishmaniasis (DCL), which is caused by *L. amazonensis* and associated with immune anergy^1,2^. The differences observed between the distinct clinical forms of TL and its associated immune activation are described to be linked to the parasite load in lesion sites^3^. In MCL lesions, parasites are rarely detected whereas in DCL lesions heavily parasitized macrophages are usually observed^2^. We have previously shown high concentrations of arginase-1 (ARG1), ornithine decarboxylase (ODC), prostaglandin E2 (PGE2) and transforming growth factor β (TGF-β) in DCL patients^4^, which could contribute to an ineffective immune response unable to hamper parasite replication. Although recent studies have shown that components of the polyamine biosynthetic pathway are linked to survival of *Leishmania sp.* inside macrophages in experimental settings^5,6^ it is unknown whether there is a differential expression of such components in patients with distinct clinical forms of TL.

Among the metabolites from the polyamine pathway, putrescine, cadaverin, spermidine and spermine are aliphatic cations derived from amino acids such as l-arginine and lysine, with multiple functions which are essential for all living organisms^7^. Polyamines are critically involved in a diverse range of cellular processes such as regulation of gene expression and translation, modulation of cell signaling, membrane stabilization and cell proliferation^7,8^. These metabolites are synthesized in a reaction catalyzed by ARG1, which converts l-arginine to l-ornithine and urea^6^. Another enzyme, ODC, catalyzes l-ornithine conversion to putrescine^6^. Putrescine then participates in an intricate cascade of reactions involving several enzymes such as spermidine synthase (SpdS) and spermine synthase (SpmS), which results in formation of polyamines, spermidine and spermine, respectively^6^. Cadaverine, a polyamine poorly studied in humans, is derived from the amino acid lysine^9^.

The uptake of l-arginine in macrophages infected with *Leishmania sp.* occurs via transporters from the cationic amino acid family (CAT)^10^. Hence, inhibition of the l-arginine transporter by melatonin reduces parasite burden by decreasing the production of polyamines^11^. We have previously demonstrated that treatment of *L. amazonensis* infected macrophages with arginase or ODC inhibitors leads to enhanced parasite clearance and dampened secretion of pro-inflammatory cytokines^4^. Indeed, different immune response profiles can influence l-arginine catabolism that, ultimately, result in resistance or susceptibility to *Leishmania* infection. l-arginine is catabolized by ARG1 in the presence of interleukin 4 (IL-4), IL-10, IL-13 and TGF-β, producing polyamines and collagen and enhancing *Leishmania* infection^12^. In converse, in the presence of pro-inflammatory mediators, such as interferon γ (IFNγ), tumor necrosis factor α (TNFα) and IL-12, the nitric oxide synthase 2 (iNOS/NOS2) will be preferentially activated, resulting in production of nitric oxide (NO) and citrulline^12,13^. Although NO alone is not sufficient to control infection, it can be further metabolized in reactive nitrogen and oxygen species, which are then involved in parasite killing^14,15^. Therefore, the profile of the host immune responses dictates differential activation of the polyamine biosynthetic pathway which strongly influences the outcome of *Leishmania* infection.

In the present study, we examined in situ (in skin lesions) and systemic concentrations of enzymes and products from the polyamine pathway in patients with LCL, MCL and DCL. We identified a distinct biosignature of DCL, with increased expression of polyamine enzymes and transporters in skin lesions and in plasma samples of DCL as compared to MCL and LCL. In addition, patients with DCL exhibited a distinct profile of gene expression in lesions. These findings suggest that the polyamine pathway contributes to disease phenotypes in tegumentary leishmaniasis.

## Results

### DCL patients exhibit high plasma levels of polyamines and amino acids

**Figure 1:**
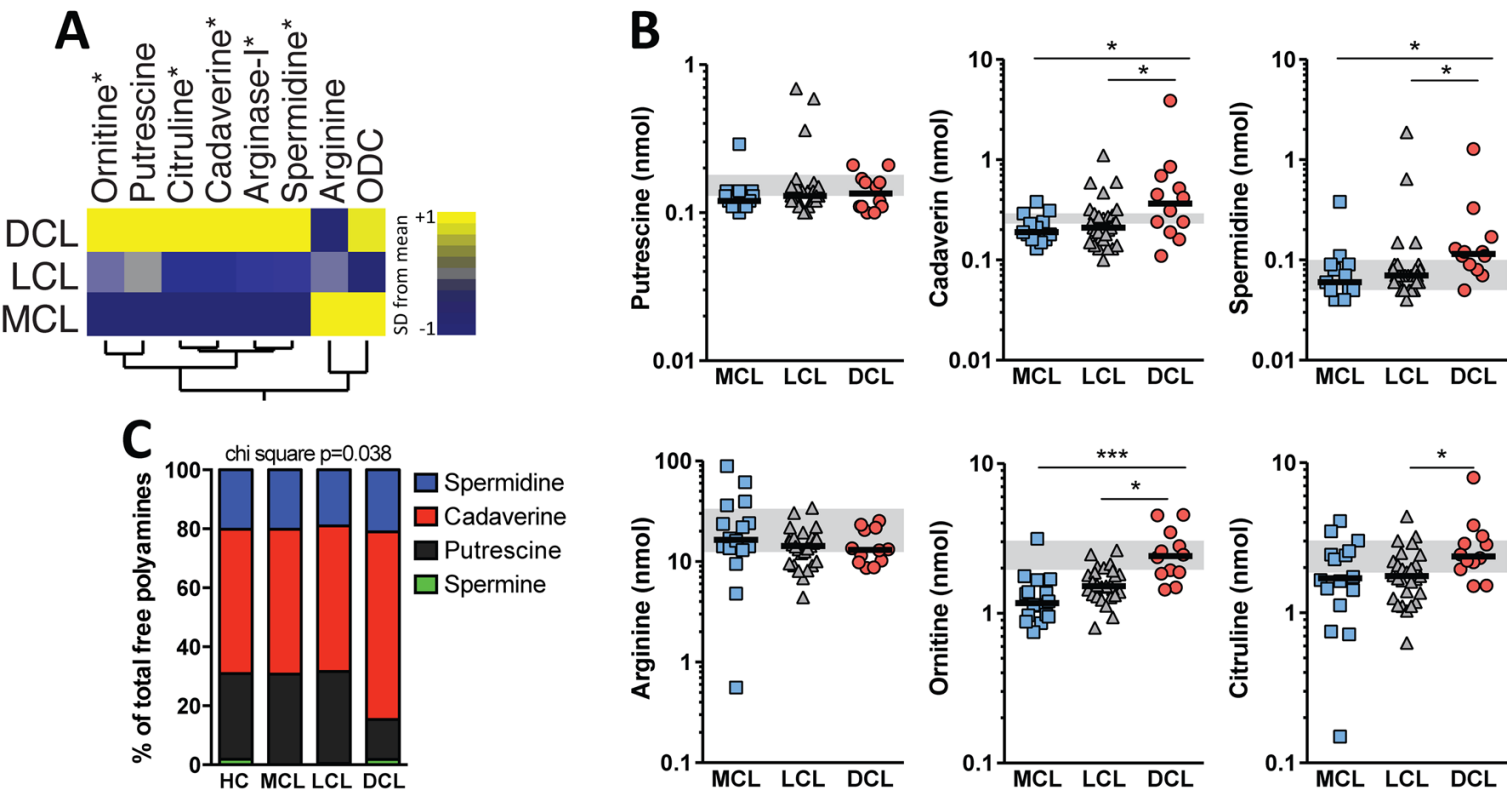
Plasma concentrations of amino acids and polyamines in patients with tegumentary leishmaniasis. Plasma levels of ARG1, polyamines: Putrescin, Cadaverine, Spermidine and amino acids: Arginine, Ornithine, Citrulline, were compared among patients with localized (LCL; n = 29), mucosal (MCL; n = 13) and diffuse (DCL) leishmaniasis as well as healthy controls (n = 43). (**A**) A hierarchical clustering analysis (Ward’s method) was employed to show the amino acids and polyamines measured using a representative profile of geometric mean values (log2 -transformed) displayed for each clinical group. The color scale of the heatmap represents z-score by row. (**B**) Univariate analyzes with scatter plots of the comparisons are shown. Data were compared using the Kruskal–Wallis test with Dunn’s multiple comparisons ad hoc test (*P < 0.05, ***P < 0.001). Lines represent median values. Grey bars represent the percentiles 25 and 75 from healthy controls (**C**) Frequency of the indicated polyamines in healthy controls or those with MCL, LCL and DCL was compared the total free polyamines using the chi square test.

Initially we tested if there is a distinct systemic profile of plasma concentrations of arginase-1 (protein), amino acids and free polyamines in patients with TL. We found that patients with DCL presented a distinct profile compared to LCL and MCL patients (Fig. 1A). We observed that the relative systemic concentrations of arginase-1, cadaverine and spermidine, but not of putrescine, were significantly higher in DCL, compared with either LCL, MCL patients or health controls (Fig. 1B, C and Table S1). Moreover, concentrations of ornithine and citrulline, but not of arginine, were significantly higher in DCL patients compared to LCL (Fig. 1B). Noteworthy, our analyses showed that, among the polyamines, cadaverine was the most abundant in DCL patients, relative to the other TL clinical forms (Fig. 1C).

### Expression of genes from the polyamine pathways in lesion biopsy specimens from patients with DCL

**Figure 2:**
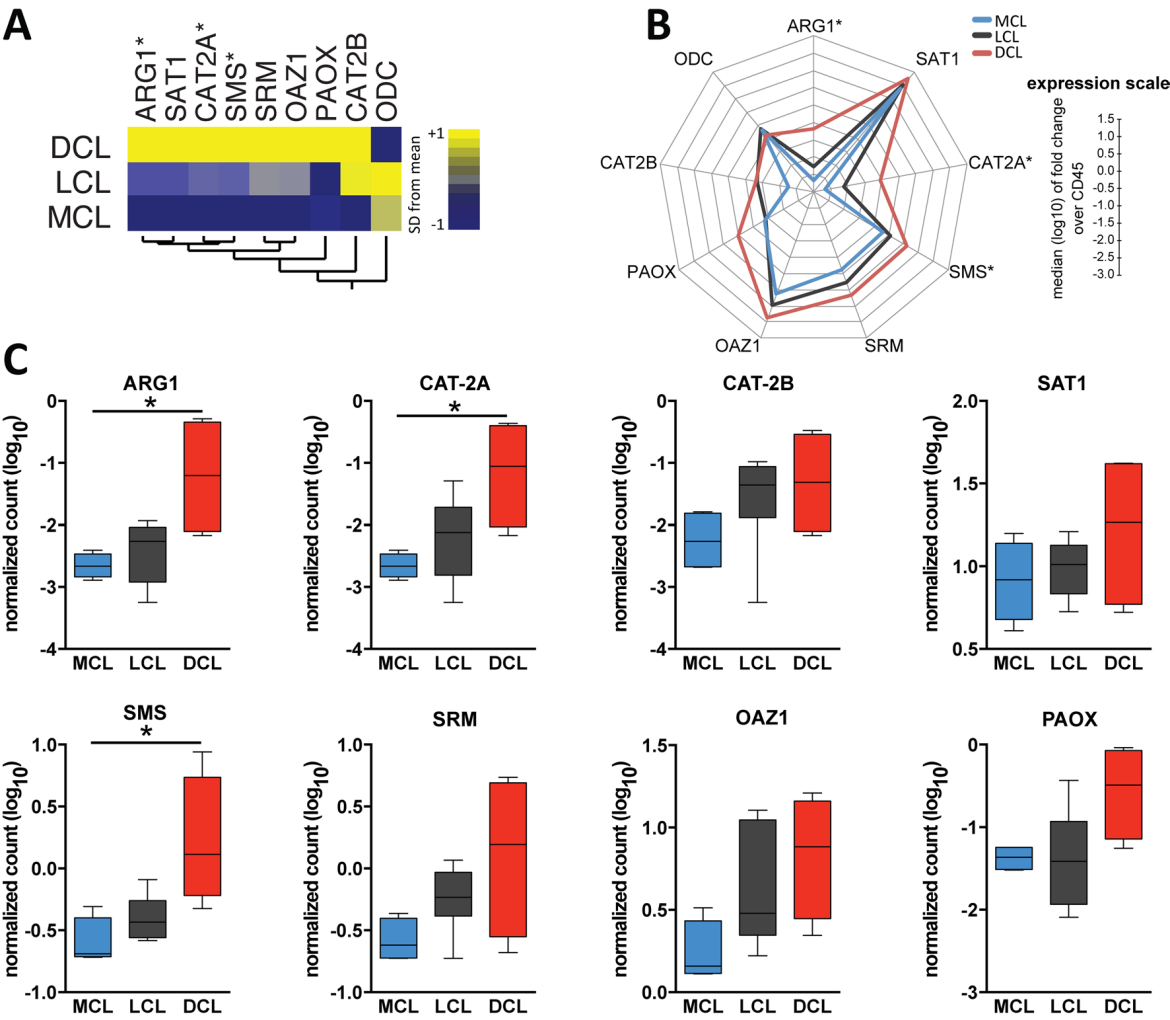
Differential expression of genes from polyamines pathways in lesions from patients with tegumentary leishmaniasis. Total RNA was extracted from lesion biopsy from patients with MCL (n = 4), LCL (n = 7) and those with DCL (n = 4). Indicated messenger RNA transcripts of host-specific cellular genes were quantified by nCounter (Nanostring) and were normalized by pan-leukocyte gene CD45 to account for detection of immune infiltration into tissues. (**A**) A hierarchical clustering analysis (Ward’s method) was employed to show the targeted genes of the polyamine pathway displaying a different profile for each clinical group. (**B**) A representative profile of geometric mean values (log2 transformed), for indicated genes, was compared among MCL, LCL and DCL patients. Data were compared using the Kruskal–Wallis test with Dunn’s multiple comparisons ad hoc test (*P < 0.05, **P < 0.01, ***P < 0.001). (**C**) Box- and-whisker plots of gene expression relative to CD45 are shown. Lines represent median values and interquartile ranges. Data were compared using the Mann–Whitney *U* test. *P < 0.05.

To further characterize the polyamine pathway in DCL, LCL and MCL patient lesions, we evaluated mRNA transcripts for key molecules isolated from skin biopsies from these clinical forms using a pre-defined nanostring panel (Table 1). We observed a specific profile of gene expression that, when combined, could successfully discriminate the different clinical groups (Fig. 2A, B). Notably, patients with DCL exhibited substantial up regulation of all genes from the polyamine pathway, except *ODC*, compared with LCL or MCL patients (Fig. 2B). Among all genes examined, we found that the expression values of *CAT2A* (isoform encoded by *SLC7A2*), *ARG1* and *SMS* were significantly higher in DCL lesions compared to MCL lesions (Fig. 2C).

**Table 1:** mRNA expression of polyamine synthetic pathways biomarkers in patient lesions with Tegumentary Leishmaniasis.

Biomarker	MCL (n = 4)	LCL (n = 7)	DCL (n = 3)	*P *value	Post-test result
ARG1	− 2.66 (− 2.83; − 2.46)	− 2.26 (− 2.9; − 1.93)	− 1.20 (− 2.10; − 0.34)	.04	c
ODC1	− 0.69 (− 0.81; − 0.54)	− 0.63 (− 0.85; − 0.32)	− 0.88 (− 1.37; 4.36)	.77	n.s
CAT2A	− 2.66 (− 2.84; − 2.47)	− 2.11 (− 2.81; − 1.71)	− 1.05 (− 2.03; − 0.4)	.04	c
CAT2B	− 2.26 (− 2.67; − 1.81)	− 1.35 (− 1.88; − 1.06)	− 1.31 (− 2.10; − 0.54)	.28	n.s
SAT1	0.91 (0.68; 1.14)	1.01 (0.83; 1.13)	1.26 (0.77; 1.62)	.68	n.s
SMS^a^	− 0.69 (− 0.71; − 0.40)	− 0.43 (− 0.56; − 0.26)	0.11 (− 0.22; 0.74)	.03	c
SRM	− 0.61 (− 0.72; − 0.40)	− 0.23 (− 0.38; − 0.03)	0.19 (− 0.55; 0.69)	.07	n.s
OAZ1	0.15 (0.11; 0.43)	0.48 (0.34; 1.04)	0.88 (0.44; 1.16)	.06	n.s
PAOX	− 1.36 (− 1.51; − 1.25)	− 1.41 (− 1.93; − 0.93)	− 0.49 (− 1.14; − 0.07)	.11	n.s

Data represent expression normalized gene count over CD45 (log2). Data was analyzed using the Kruskal–Wallis test with Dunn’s multiple comparisons ad hoc test. Comparisons with *P *value < .05: LCL X MCL, LCL X DCL, ^a^ DCL X MCL; n.s. nonsignificant. *MCL* mucosal cutaneous leishmaniasis; *LCL* localized cutaneous leishmaniasis, *DCL* diffuse cutaneous leishmaniasis, *ARG1* Arginase 1, *ODC* ornithine decarboxylase, *CAT2A* cationic aminoacid transporter 2A, *CAT2B* cationic aminoacid transporter 2B, *SAT1* spermidine-spermine acetyl trabtransferase, *SMS* spermine synthase, *SRM* spermidine synthase, *OAZ1* ornithine antyenzime, *PAOX* peroxisomal oxidase.

### Transcriptomic analyses of tegumentary lesions from publicly available datasets validate the differential expression of the polyamine biosynthetic pathway in distinct clinical forms of leishmaniasis

**Figure 3:**
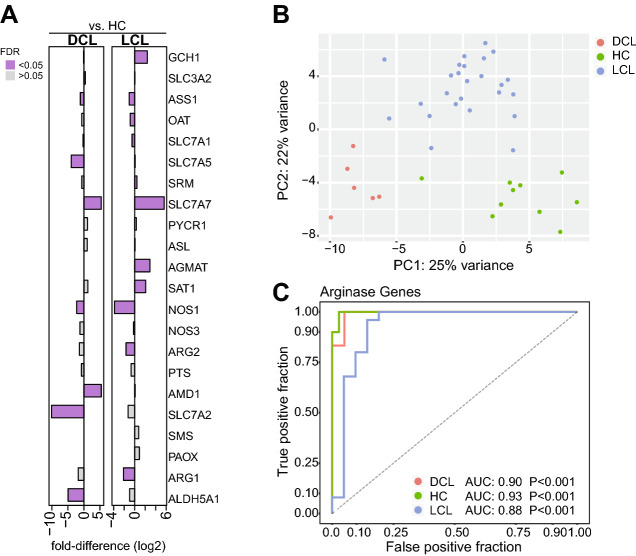
Tegumentary leishmaniasis patients display a unique profile of gene expression from the polyamine biosynthetic pathway. (**A**) Genes involved in the polyamine pathway were retrieved from the transcriptome dataset as described in “Methods” section and used in additional analyses to test whether its expression values were able to separate the distinct clinical groups. Fold-differences (DCL or LCL vs. healthy controls) were calculated and statistically significant differences are highlighted in colored bars. (**B**) A principal component analysis (PCA) model was employed to verify if the expression values of the genes from the polyamine biosynthetic pathway were able to classify the samples within the groups, regardless of the fact that some of these genes were not differentially expressed between the clinical groups. We used the normalized count table without the calculation of fold difference against the control group to input the PCA algorithm (see “Methods” section for details). (**C**) A Receiver Operator Characteristics (ROC) curve analysis was performed with these same gene expression values, to assess the sensibility and specificity of this classification. The ROC curve analysis used a multinomial model, in which the outcomes (HC, DCL and LCL) were binarized. Thus, this approach allowed us to compare the power of all the genes from polyamine biosynthetic pathway described above to discriminate between the following groups: (i) DCL vs. HC + LCL; (ii) HC vs. LCL + DCL; and (iii) LCL vs. HC + DCL. AUC: area under the curve.

To validate our findings on the polyamine pathway in TL, we re-analyzed the gene expression data from an independent patient cohort, which was recently published^16^. This approach revealed that skin lesions from TL patients generally exhibit a distinct gene expression profile compared to normal skin from uninfected healthy endemic controls (Fig. 3A). Fold-difference analysis highlighted that DCL patients exhibited higher expression of *AMD1*, whereas *SLC7A2* was down modulated compared to health controls (Fig. 3A). Additional investigation using a principal component analysis (PCA) model revealed that the overall gene expression profile of the transcripts evaluated here was able to effectively segregate the TL clinical forms (Fig. 3B, C). Such PCA model including expression values of all genes validated the results from the fold-difference analysis, with segregation of all clinical forms (Fig. 3B). Indeed, a discriminant analysis using ROC curves of such combination of genes resulted in high accuracy in distinguishing the clinical groups (Fig. 3C).

**Figure 4:**
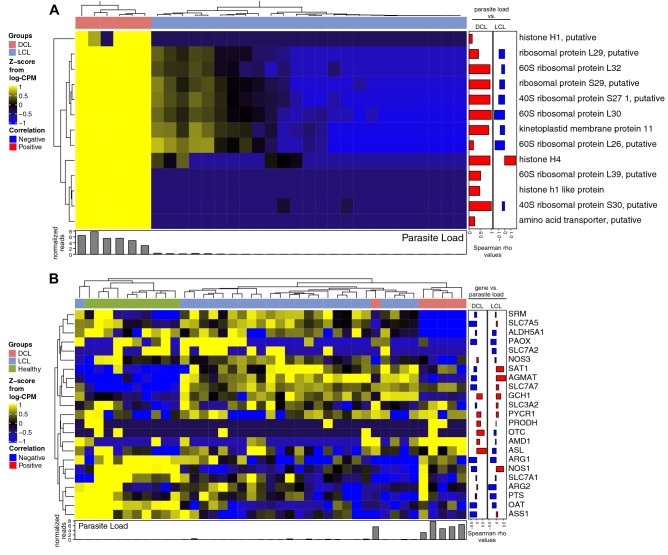
Association between activation of genes from the polyamine pathway and parasite transcripts. (**A**) A hierarchical clustering analysis (Ward’s method) was employed to illustrate the overall expression profile of *Leishmania* genes in DCL and LCL lesion and its correlation with parasite loads (normalized leishmania transcripts as described in “Methods” section). Identifiers refer to putative functions (described in “Methods” section). (**B**) A similar statistical approach was used to evaluate the overall profile of genes of the polyamine biosynthetic pathway in TL patients vs. parasite loads. Correlations were tested using the Spearman rank. In (**A**) and (**B**), each column represents one patient. Spearman correlation rank coefficient (rho) values were displayed as bar plots, to demonstrate strength and directionality of the associations.

We next used the same dataset described above to examine if the expression profile of the genes from the polyamine pathway was associated with parasite load in patient lesions. In fact, the estimated values of parasite load were correlated with: (i) the *Leishmania* genes (which included annotated genes and also putative genes) (Fig. 4A) and (ii) host genes from the polyamine biosynthetic pathway (Fig. 4B). Hierarchical cluster analysis of the *Leishmania* transcripts quantified by the RNAseq indicated that patients with DCL were the ones who displayed the highest levels of parasite transcripts (Fig. 4A). This approach revealed in Fig. 4A that expression values of all the *Leishmania* genes examined were positively correlated with parasite load in the group of patients with DCL, but not in those with LCL. Indeed, only histone H4 expression level displayed a positive correlation with the parasite load in the group of LCL patients whereas levels of histone H1 putative, 60S ribosomal protein L39 putative, histone h1 like protein, and amino acid transporter (putative) were not statistically correlated and the remaining genes from the list were actually negatively correlated with parasite loads. Finally, we tested if the expression values of genes from the polyamine pathway could be associated with parasite load. First, the overall gene expression profile of the host polyamine pathway could separate the individuals from the distinct clinical groups (Fig. 4B), corroborating with the results from the PCA model (Fig. 3B). Our analyses also demonstrated that while expression values of several genes exhibited similar association profiles with parasite loads between DCL and LCL patient groups, such as *ARG1*, other targets displayed a divergent pattern, such as *NOS1* and *NOS3* (Fig. 4B). Intriguingly, the analyses of a publicly available transcriptome dataset of skin samples demonstrated that expression values of both *ARG1* and *ARG2* were higher in healthy controls than in LCL (Fig. 3A). In addition, the expression of these molecules was slightly lower, but not statistically significant in DCL patients compared to healthy controls. Furthermore, the transcriptome data also indicated that expression values of *ARG1* were inversely correlated with parasite loads in the lesions from both LCL and DCL whereas the those of *ARG2* were also negative correlated in LCL but only marginally directly correlated with parasite loads in DCL lesions (Fig. 4B).

## Discussion

The polyamine biosynthetic pathway has been described as critical to promote *Leishmania* intracellular replication inside host macrophages^6,18,19^. In this exploratory study, we performed a detailed investigation of the expression profiles of components of such pathway in skin lesions and in peripheral blood of patients presenting with distinct clinical forms within the spectrum of TL disease.

Our analyses demonstrated that DCL patients, compared with LCL and MCL, presented higher plasma concentration of polyamines (spermidine and cadaverin) and specific amino acids (ornithine and citruline), identifying a distinct biosignature that characterized this anergic form of TL. In mammalian cells, putrescine is the precursor for spermidine formation and probably for this reason its concentrations are usually low^18–20^. Indeed, we did not find any difference in the plasma concentrations of putrescine between the clinical forms, although spermidine concentrations were higher in DCL patients. We hypothesize that putrescine may be rapidly converted into other polyamines that promote *Leishmania* replication in DCL lesions. Interesting, cadaverin was the most abundant polyamine detected in plasma from the TL patients, and especially in those with DCL. Previous studies reported that cadaverin is induced to compensate significant decreases in concentrations of polyamines or their substrates^21^. Studies with *Escherichia coli* cell-free system showed that cadaverin has the same ability as putrescine and spermine in promoting protein synthesis^21^. The role of cadaverin in parasitic infections is still largely unknown.

Previous work from our group has shown that patients with DCL display elevated plasma protein concentrations of ARG1 and ODC^4^. These enzymes regulate arginine availability in through regulation of its precursor, ornithine^22^. Arginase is also involved in the urea cycle, in which it catalyzes interconversion of arginine-citrulline-ornithine^22^. l-arginine is considered a conditionally essential amino acid because even though it is synthesized in human body, it still needs dietary supplementation^23^. Interestingly, here we demonstrate that, although arginine plasma concentrations were not different among the distinct TL clinical forms, DCL patients had high concentrations of ornithine and citrulline. Although citrulline can be produced from arginine by iNOS/NOS2^22^, our findings led us to suggest that in the context of DCL, this amino acid is potentially being interconverted by arginase, favoring the production of polyamines.

In recent years, there has been an increase in the number of studies describing the importance of amino acid transporters in the trypanosomatid metabolism and especially in *Leishmania*. Most amino acids are involved in osmotic control, metacyclogenesis, establishment of infection, regulation of autophagy and apoptosis, resistance to oxidative stress, and synthesis of polyamines^24^. Regarding the essential amino acids for the biosynthesis of polyamine, such as arginine and ornithine, *Leishmania* is auxotrophic for arginine and depends on uptake from the external environment through a specific transporter, amino acid permease 3 (AAP3)^17,25,26^. This means that the production of polyamines in the parasites is directly related to their ability to acquire such amino acids from the host cell^26^. In fact, *Leishmania sp*. has been described to benefit from host-derived polyamines and several studies have indicated that genetic or pharmacological suppression of parasite enzymes involved in polyamine pathways results in impairment of parasite growth and establishment of host cell infection^22,27,28,29^. More recently, it has been shown that during infection, *L. amazonensis* is able to alter the host metabolism, inducing polyamine production^30^. Although in our analyses we could not find a specific AAP3 gene, due to the lack of genome annotation, we retrieved the functional annotation in other *Leishmania* putative genes that are involved amino acid transport (shown in Fig. 4 as amino acid transporter, putative). Our results demonstrated a higher expression of the parasite amino acid transporter (putative) in DCL lesions, which positively correlated with the total counts of parasite transcripts, reinforcing the hypothesis that higher expression of amino acid transporters by *Leishmania* may indeed favor parasite persistence during infection. It is possible that the systemic balance of polyamines and amino acids induced by infection could contribute to the persistence of the high parasite loads observed in DCL lesions. Our results are in agreement with this idea, although a formal confirmation by mechanistic studies is still necessary.

In the present study, we tested if there are differences in expression of the target genes of the polyamine pathway among different clinical forms in skin lesions. Our results demonstrated that among the expression values of *ARG1, CAT-2A* and *SMS* were higher in DCL patients. It has been shown that *CAT-2* expression values are increased by both *Leishmania* infection and l-arginine deprivation^10^. Therefore, we hypothesize that parasites from DCL patients positively regulate *CAT-2A* expression, thereby increasing polyamine production, which is important for their proliferation and maintenance of infection. In previous studies from our group and others, lesions from DCL patients were found to present greater expression of IL-4 and IL-10 transcripts in relation to TNF expression^4^, whereas lesions from patients with LCL and MCL are characterized by high expression of IFNγ mRNA and absence of IL-4^3^. In addition, THP-1 macrophages infected with *L. donovani* are described to exhibit increased l-arginine uptake by CAT-2, augmented ARG1 activity and higher levels of spermidine, that correlated with increased concentrations of IL-10 and reduced concentrations of IL-12 and TNF-α^10^. Whether the increased expression of *ARG1* and *CAT-2A* in skin lesions from patients with DCL is associated with the immunosuppression warrants further investigation.

The analysis in patient lesions demonstrated that *ODC1* was not a part of the biosignature of the polyamine pathway in DCL skin lesions. ODC is the key enzyme implicated in polyamine biosynthesis required for in vitro intracellular proliferation in *Leishmania*-infected cells^19,31^. The intracellular concentration of polyamines is regulated by several mechanisms, including the synthesis, degradation and efflux/uptake by the polyamine transporters^6^. High concentrations of intracellular polyamines induce the expression of important enzymes involved in polyamine recycling: spermidine/spermineN1-acetyltransferase (SSAT) and peroxisomal n(1)-acetyl-spermine/spermidine oxidase (PAOX), associated to interconversion and degradation of polyamines. Another important step of the regulation of polyamine production is mediated by the fast turnover of ODC. When cellular polyamine levels are high, they induce the biosynthesis of an ODC inhibitor named ODC antizyme (OAZ), which prevents its dimerization and promotes ODC degradation, through the 26S proteasome^32,33^. OAZ is induced by an excess of polyamines (and has a fast turnover), and besides regulating the degradation of ODC, negatively regulates cellular polyamine transporters^34^. However, the proteins involved in polyamine transport and the exact mechanisms by which polyamines regulate their uptake in the mammalian cells are not well known. Cells in which the OAZ enzyme is expressed to high levels exhibit a marked reduction in polyamine uptake. Our data suggest that high expression of *OAZ* in the DCL lesion could be responsible for the degradation of ODC, justifying the low levels of this enzyme. However, the exact molecular and cellular mechanisms involved in *ODC* in lesions or systemic regulation in DCL patients remain to be explored.

Although the RNA transcripts for *CAT-2B, SSAT1, SRM, OAZ* and *PAOX* did not show statistical difference between the diverse TL clinical forms (Fig. 2C), we observed a distinct biosignature of the polyamine biosynthetic pathway in DCL individuals in the hierarchical cluster analysis, corroborating the idea that this metabolic pathway is indeed important in parasite proliferation and successful establishment of *Leishmania* infection. However, a limitation of the present study was that we did not have available sample/data to directly test correlations between polyamine expression and parasite burden in the lesions. Thus, we employed an analytical approach to specifically test this hypothesis in a separate patient cohort.

An important contribution of our study is the validation analysis using an independent patient cohort recently published^16^. In this approach, we tested whether the transcriptomic profile also revealed differential gene expression in the TL disease groups. Indeed, the transcriptional data from skin biopsies from DCL and LCL patients compared to normal skin revealed that the targets from the polyamine pathway are able to discriminate the distinct TL clinical groups. Interestingly, the *SLC7A2* gene was down modulated in DCL patients. Arginine transport is mediated by members of the solute carrier 7 family (SLC7), that is divided into two subfamilies: the cationic amino acid transporters (CATs; *SLC7A1-4* and *SLC7A14*) and L-type amino acids transporters (LATs: *SLC7A5-13*), also known as CATs^35^. Although expression of this gene diverges between the RNAseq and *n-counter* analyses, being higher in the first and lower in the latter, CAT2A and CAT2B are isoforms of the *SLC7A1* gene and by RNAseq technique, we could not identify such isoforms separately. According to our study, the Adenosylmethionine decarboxylase 1 (*AMD1*) up regulation in DCL patients may be relate to the high concentrations of spermidine found in these patients. AMD1 is an enzyme involved in biosynthesis of spermidine^36^. The importance of this enzyme in tumorigenesis in gastric cancers associated with polyamine synthesis has been recently been demonstrated^36^. Of note, its role in parasitic diseases, including *Leishmania* infection, is still not clear.

The transcriptome data from skin lesions indicated that expression levels of *ARG1* and *ARG2* were not statistically distinct between DCL patients and healthy controls. Further analyses also revealed a negative correlation between *ARG1* expression levels and parasite loads in both LCL and DCL whereas *ARG2* expression was negatively correlated in LCL but slightly positively correlated with parasite loads in DCL lesions. At first glance, such findings may contrast with the idea that arginase-1 is involved in the pathogenesis of DCL, as previously reported^4^. Our experiments in plasma revealed higher levels of arginase-1 in DCL patients compared to controls, and the nanostring experiment unfortunately did not include samples from healthy controls for comparisons. Although the results presented here clearly show a differential modulation of the polyamine pathway in distinct clinical forms of TL, additional studies are still warranted to investigate the direct participation of arginase-1 in cellular responses to *Leishmania* in skin lesions.

The transcriptome analyses also revealed that the overall gene expression profile of the polyamine pathway is associated with the parasite loads and that such association is linked to the TL clinical presentation (in either LCL or DCL). Although the expression level of genes from the parasite should be proportional to transcriptionally estimated parasite load, it is known that post-transcriptional, epigenetic regulation can result in discrepancies between abundance of a given gene and number of parasites. The previous report on this transcriptome data has identified the 15 most highly and uniformity expressed parasite genes in each of the patients analyzed with DCL^16^. It is indeed expected that such expression levels of genes should directly follow the parasite loads. However, we detected an important difference in the profile of correlation between expression levels of *Leishmania* genes and parasite loads between the groups of DCL and LCL. This finding is surprising and reinforce the hypothesis of differential modulation of gene expression occurring in the parasites. Interestingly, among the genes that exhibited divergence in association of expression levels with parasite loads between DCL and LCL are those whose functions are related to amino acids transport. *Leishmania* putative genes of these transporters were shown here to be highly expressed in patients with DCL and the expression level was related to parasite loads in DCL but not in LCL, supporting the hypothesis that parasites may take advantage of an immunomodulated environment. Additional studies are necessary to test if the activation of the polyamine pathway is a cause or consequence of the higher parasite loads in DCL.

**Figure 5:**
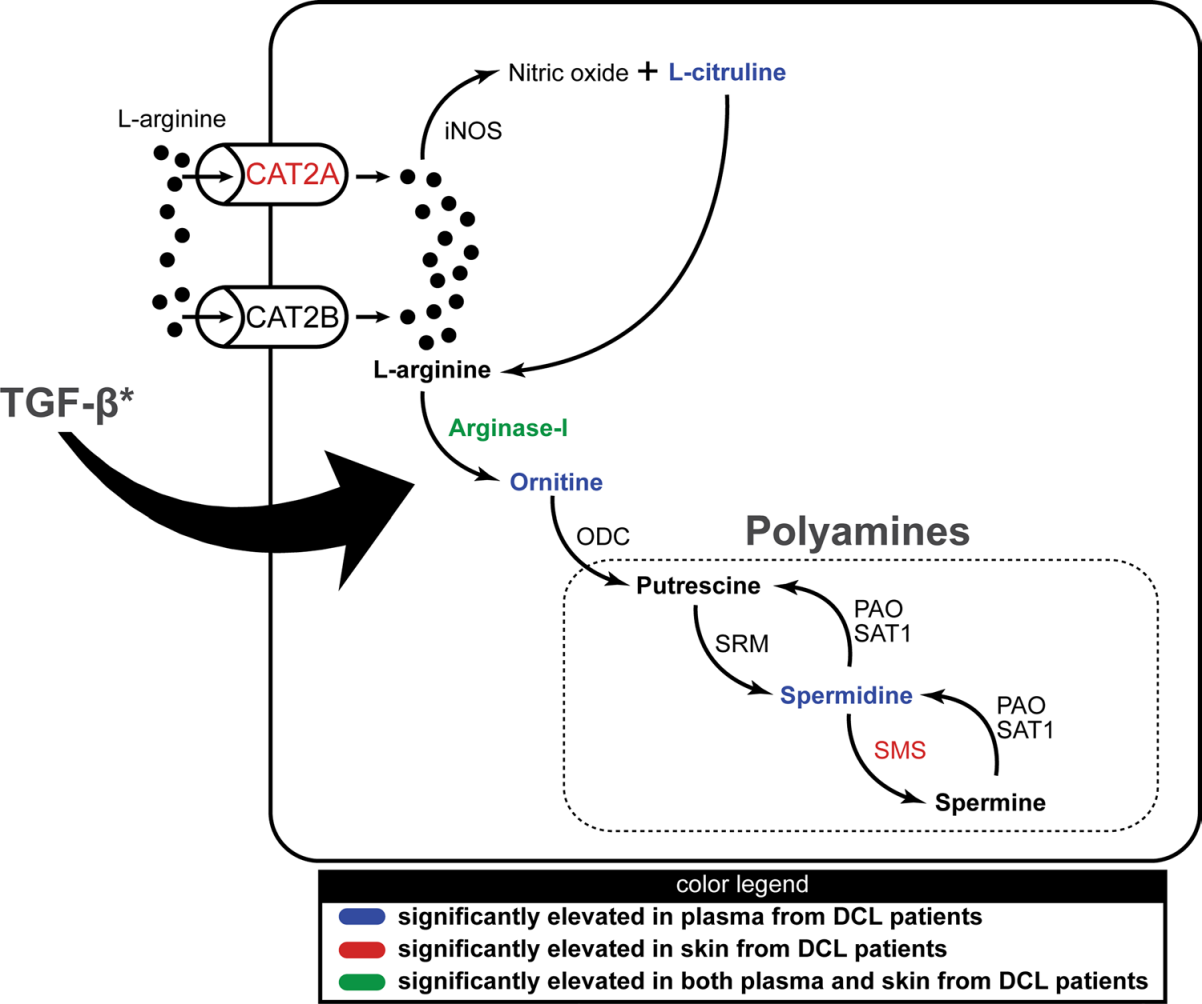
Polyamine pathway in patients with Diffuse Cutaneous Leishmaniasis. This illustration summarizes the data of Figs. 1 and 2. The figure shows the cascade of the polyamine biosynthesis and highlights the parameters which were statistically significant in plasma and/or skin lesions of Diffuse Cutaneous Leishmaniasis (DCL) patients compared to the other clinical forms of tegumentary leishmaniasis in our study settings.

In conclusion, our findings indicate that the differential activation of the polyamine pathway characterizes DCL relative to other clinical forms of TL (summary of findings from the experiments performed here are illustrated in Fig. 5) and open perspectives for future studies testing the manipulation of such pathway to reduce immunopathology in TL.

## Methods

### Ethics statement

This study was approved by the institutional review board from Instituto Gonçalo Moniz, Fundação Oswaldo Cruz. All clinical investigations were conducted according to the Declaration of Helsinki. Written informed consent was obtained from all participants or legal guardians, and all data analyzed were anonymized.

### Study design

We performed a cross-section study of patients presenting with different clinical forms of TL as well as uninfected endemic controls. Social-demographic and clinical characteristics of all patients were previously described^4,37^. DCL cases (n = 14) were obtained from a study performed between 1980 and 1990 in the state of Maranhão, Northeast of Brazil^4^. DCL diagnosis was based using previously described criteria^1,2^. In addition, we evaluated data from age- and sex-matched patients with MCL (n = 13) or LCL (n = 29) recruited at our reference clinic in Jiquiriçá, BA-Brazil, as previously described^37^. MCL and LCL individuals included in the present study were required to have no previous history of TL and to be treatment naïve. For plasma analyses, we included samples from 43 healthy controls (matched by age and sex to the TL groups) from the same region of endemicity and who had negative results of an anti-*Leishmania* delayed-type hypersensitivity test. Diagnosis criteria for LCL and MCL were published previously^37^ and were based on anti-*Leishmania* delayed-type hypersensitivity test, detection of anti-*Leishmania* antibody, or detection *of Leishmania* parasites in biopsy tissue specimens by either immunohistochemistry or qualitative polymerase chain reaction (PCR) assays.

### Polyamine trofiles

The polyamine profiles and content were performed according to a previous study^38^ Briefly, serum samples from patients with Leishmaniasis (60 µl) were mixed with cold 5% (v/v) perchloric acid at a ratio of 1:4 (v/v). Then, the samples were submitted to three cycles of freezing (− 20 °C) and thawing (at room temperature), prior to centrifugation at 11,000*g* for 10 min. The supernatant containing free polyamines was collected. Free polyamines were derivatized with dansylchloride (5 mg ml^−1^ in acetone), 0.05 mM diaminoheptane-DAH (internal standard) and saturated sodium carbonate. After 50 min incubation in the dark at 70 °C, the excess of dansylchloride was converted to dansylalanine by adding proline (100 mg ml^−1^). After 30 min incubation (room temperature), dansylated PAs were extracted with of toluene 1:1 (v/v), the organic phase containing the polyamines was collected. The toluene phase was evaporated with gaseous nitrogen (40 °C). Dansylated polyamines were resuspended in 100 µl of acetonitrile.

Polyamines were separated by high-performance liquid chromatography (HPLC, Shimadzu, Japan) using a C18 reversed-phase column (5 µm × 4.6 mm × 250 mm—Sulpelcosil, Supelco), as described previously^38^. Polyamines were detected at 340 nm (excitation) and 510 nm (emission) wavelengths with an RF-20A fluorescence detector (Shimadzu). Peak areas and retention times were measured by comparison with standard known concentrations of polyamines (Table S1).

### Free amino acids profiles

The amino acid content was determined as previously described^39^. Serum samples from patients with Leishmaniasis (60 µl) were extracted in 1.8 ml of 80% ethanol (v/v) and concentrated in ‘speed vac’. Samples were resuspended in 0.6 ml Milli’Q and centrifuged at 20,000*g* for 10 min. The supernatant was filtered through a 20 µm membrane. Amino acids were derivatized before injection with o-phthalaldehyde and separated by HPLC (Shimadzu) on a C18 reverse phase column (as described above). The gradient program was developed as in^40^, with a total running time of 55 min (including the time for column regeneration) at a flow rate of 1.0 ml min^−1^, at 40 °C. Mobile phase A is a 0.1 M sodium acetate pH 7.2 and Mobile phase B is 100% methanol (MeOH). The proportion of mobile phase B (MeOH 100%) is as follows: 0–15 min, 14%; 15–20 min, 30%; 20–24 min, 35%; 24–26 min, 47%; 26–34 min, 50%; 34–38 min, 70%; 38–40 min, 100%; 40–45 min, 100%; 45–55 min, 14%; and 55 min, 14%. A fluorescence detector (Shimadzu, RF-20A), set at 250 nm excitation and 480 nm emission wavelengths, was used for detection and quantification. Peak areas and retention times were measured by comparison with standard known concentrations of amino acids.

### Arginase-1 protein measurement in plasma

Plasma levels of arginase 1 (Hycult Biotech, Uden, the Netherlands) were measurement using enzyme-linked immunosorbent assay ELISA according to the manufacturer’s instructions.

### nCounter analysis

Tissue samples from which we had high-quality messenger RNA (mRNA) were obtained from a subset of three patients with DCL, four patients with MCL and seven patients with LCL who were also matched for age and sex. Skin tissues were used from DCL and LCL whereas nasal mucosal samples were obtained from MCL patients as previously described^4,37^. Total RNA was extracted from cryopreserved lesion biopsy specimens, using Trizol reagent (Invitrogen, Carlsbad, California), with an additional purification step using RNeasy columns (Qiagen, Venlo, Netherlands). nCounter analysis (NanoString Technologies, Seattle, Washington) was performed based on direct molecular bar coding of target RNA transcripts and digital detection^41^. The chosen targets genes were: *ARG1* (Arginase 1), *ODC1* (Ornitine Decarboxylase 1), *SRM* (Spermidine Syntahse), *SMS* (Spermine Synthase), *SAT1* (spermidine-spermine acetyl transferase), *PAOX* (Peroxisomal oxidase), *SLC7A2* (CAT2-cationic aminoacid transporter 2), *OAZ1* (Ornithine Decarboxylase antienzyme). To account for differences in leukocyte infiltration between patient lesions, data were normalized for CD45, which encodes the pan-leukocyte marker CD45, detectable at fentomolar range, as previously reported^41^.

### RNA-seq analysis

Data samples were downloaded from *bioproject PRJNA307599* and labeled according to the informed metadata (41 samples sequenced with *Illumina HiSeg 2000* of 10 controls, 8 early LCL biopsies 17 late LCL biopsies and 6 DCL biopsies with unspecified disease duration). For all samples, low-quality bases have been removed and adapters were trimmed using *Trimmomatic* V0.32^42^. After quality check, sequences were aligned to the human reference transcriptome (GRCh38.p13—https://www.ncbi.nlm.nih.gov/assembly/GCF_000001405.26/ version), comprising both mRNA and miRNA transcripts, and the recent versions of *L. braziliensis* (MHOM/BR/75/M2903) and *L. major Fiendlin* transcriptomes obtained from the *TriTrypDB* database (www.tritrypdb.org), with *Salmon* (v0.8.2)^43^. After mapping, the *Salmon* output was converted with the *tximport* package to count table by R (3.5.3 version). We used the Reactome database (R-HSA-351202), to identify the genes involved in the polyamine biosynthetic pathway. Using this approach, we retrieved 22 genes from the transcriptome dataset: *GCH1, SLC3A2, ASS1, OAT, SLC7A1, SLC7A5, SRM, SLC7A7, PYCR1, ASL, AGMAT, SAT1, NOS1, NOS3, ARG2, PTS, AMD1, SLC7A2, SMS, PAOX, ARG1* and *ALDH5A1* (as shown in Fig. 3A) and they were used in additional analyses to test whether its expression values were able to separate the distinct clinical groups. Differentially expressed genes (DEGs) were examined by *edgeR* package^44^. The parasite burden from RNAseq data was measured by normalizing the library size^45^. The gene expression values used were the TMM-normalized log CPM values. The correlation between the parasite burden and gene expression values (both human and leishmania) were performed with the *Hmisc* package. As only a few *Leishmania* genes have been functionally annotated by biological assays, we collapsed the genes by putative functions and used the most representative expressed genes (Table S2). This strategy was employed to avoid the synonymies between genes with the same function; such approach is commonly used in high throughput analysis (https://www.ncbi.nlm.nih.gov/pmc/articles/PMC3166942/pdf/1471-2105-12-322.pdf). Changes in gene expression were considered significant when statistical test values (false discovery rate [FDR] adjusted p-value) were lower than 0.05 and the fold-difference higher than ± 1.5. Principal Component Analysis (PCA) was performed using the TMM-normalized log-transformed CPM values with *plotPCA* function from *Deseq2* package^46^ to verify if the expression values of the genes from the polyamine biosynthetic pathway were able to classify the samples within the groups, regardless of the fact that some of these genes were not differentially expressed between the clinical groups. The receiver operator characteristics (ROC) curves with area under the curve were measured and plotted with *pROC* package^47^. The ROC curve analysis was performed with the same gene expression values which were inputted in the PCA algorithm, to assess the sensibility and specificity of this classification. Moreover, the ROC curve analysis used a multinomial model, in which the outcomes (HC, DCL and LCL) were binarized. Thus, this approach allowed us to compare the power of all the genes from polyamine biosynthetic pathway described above to discriminate between the following groups: (i) DCL vs. HC + LCL; (ii) HC vs. LCL + DCL; and (iii) LCL vs. HC + DCL. Heatmaps of the polyamine pathway genes as well as of the Leishmania genes were plotted with the *Complexheatmap* package^48^.

### Data analysis

Median values with interquartile ranges (IQRs) were used as measures of central tendency and dispersion. For expression assays, Mann–Whitney *U* test was used to compare the groups. Plasma values were compared using Kruskal–Wallis test with the Dunn’s multiple comparisons test. Unsupervised 2-way hierarchical cluster analyses (Ward’s method) with bootstrap were used to test whether patients with DCL, MCL and LCL can be grouped separately on the basis of simultaneous quantification from their plasma and lesion polyamine biosynthetic pathway profile. Dendrograms represent Euclidean distance. Differences with *p *value < 0.05 were considered statistically significant.

## Supplementary information

Supplementary file1 (PDF 138 kb)
