# Supplementary material for: Differential expression of polyamine biosynthetic pathways in skin lesions and in plasma reveals distinct profiles in diffuse cutaneous leishmaniasis
Source: Sci Rep. 2020 Jun 29;10:10543. doi: 10.1038/s41598-020-67432-5 (PMC7324605; doi:10.1038/s41598-020-67432-5)
Supplement: Supplementary file 1 — Supplementary file1 (PDF 138 kb) [file 41598_2020_67432_MOESM1_ESM.pdf]

Differential expression of polyamine biosynthetic pathways in skin lesions and in plasma reveals distinct profiles in diffuse cutaneous leishmaniasis

Hayna Malta-Santos[1,2*]; Jaqueline França-Costa[1, 2*]; Amanda Macedo[3]; Artur T. L. Queiroz[2]; Kiyoshi F. Fukutani[2,4]; Sandra Marcia Muxel[5]; Ricardo Khouri[1,2]; Johan Van Weyenbergh[2,6]; Viviane Boaventura[1,2]; Aldina Barral[1,2]; Jackson M. Costa[2.7]; Eny Iochevet Segal Floh[3]; Bruno B. Andrade[1,2,4,8,9]; Lucile M. Floeter-Winter[5]; Valéria M. Borges[1,2] #

[1]Universidade Federal da Bahia, Salvador, Brazil

[2]Instituto Gonçalo Moniz (IGM), Fundação Oswaldo Cruz (FIOCRUZ), Salvador, Brazil

[3]Departamento de Botânica, Instituto de Ciências Biomédicas, Universidade de São Paulo, São Paulo, Brazil

[4]Multinational Organization Network Sponsoring Translational and Epidemiological Research (MONSTER), Salvador, Brazil

[5]Departamento de Parasitologia, Instituto de Ciências Biomédicas, Universidade de São Paulo, São Paulo, Brazil

[6]Department of Microbiology and Immunology, Rega Institute for Medical Research, University of Leuven, Belgium

[7]Universidade Federal do Maranhão, São Luis, Brazil

[8]Escola Bahiana de Medicina e Saúde Pública, Salvador, Brazil

[9]Universidade Salvador (UNIFACS), Laureate Universities, Salvador, Brazil

#Corresponding author: vborges@bahia.fiocruz.br (VMB).

*These authors equally contributed to the work

Supplementary Table 1

Table 1. Systemic expression of ARG1 and ODC enzymes and free polyamines in Tegumentary Leishmaniasis

Biomarker (pg/ml)	HC (n=40)	MCL (n=14)	LCL (n=29)	DCL (n=12)	*p*-Value	Post- test result
ARG1	0.13 (0.087;0.2)	0.42 (0.12; 1.53)	3.36 (2.67; 5.18)	18.03 (11.53; 30.96)	<.0001	a,c,d,e
ODC	318.5 (119.3; 550.8)	577.0 (215.0; 1072)	242.0 (126.0; 389.0)	556(469.3; 847.5)	.0064	b,e
PUT	0.15 (0.13; 0.18)	0.12 (0.11; 0.14)	0.13 (0.12; 0.15)	0.13 (0.10; 0.19)	.0096	f
SMS	nd	nd	nd	nd	N/A	N/A
SRM	0.07 (0.05; 0.1)	0.06 (0.04; 0.09)	0.07 (0.06; 0.08)	0.11 (0.08; 0.16)	.02	c
CAD	0.29 (0.23; 0.38)	0.19 (0.16; 0.09)	0.21 (0.15; 0.28)	0.36 (0.20; 0.64)	.007	n.s.

Data was analyzed using the Kruskal-Wallis test with Dunn's multiple comparisons ad hoc test. Comparisons with P-value <.05: [a]LCL X MCL, [b]LCL X DCL, [c] DCL X MCL, [d] HC X LCL , [e] HC X DCL, [f] HC X MCL; n.s. nonsignificant. HC, Healthy Control; MCL, Mucosal Cutaneous Leishmaniasis; LCL, Localized Cutaneous Leishmaniasis; DCL, Diffuse Cutaneous Leishmaniasis. ARG1, Arginase 1; ODC, ornithine decarboxylase; PUT, Putrescin; SMS, spermine synthase; SRM, spermidine synthase; CAD, cadaverin.

Supplementary Table 2

gene	function
LmjF.27.1190:mRNA	histone h1 putative
LmjF.27.1240:mRNA	histone h1 putative
LmjF.36.3390:mRNA	ribosomal protein L29, putative
LmjF.36.3400:mRNA	ribosomal protein L29, putative
LmjF.35.2050:mRNA	60S ribosomal protein L32
LmjF.21.1720:mRNA	60S ribosomal protein L32
LmjF.28.2460:mRNA	ribosomal protein S29, putative
LmjF.28.2205:mRNA	ribosomal protein S29, putative
LmjF.36.3750:mRNA	40S ribosomal protein S27-1, putative
LmjF.35.0240:mRNA	60S ribosomal protein L30
LmjF.35.2210:mRNA	kinetoplastid membrane protein-11
LmjF.35.2220:mRNA	kinetoplastid membrane protein-11
LmjF.24.2050:mRNA	60S ribosomal protein L26, putative
LmjF.35.1670:mRNA	60S ribosomal protein L26, putative
LmjF.02.0020:mRNA	histone H4
LmjF.31.3180:mRNA	histone H4
LmjF.35.1310:mRNA	histone H4
LmjF.36.0020:mRNA	histone H4
LmjF.29.2370:mRNA	60S ribosomal protein L39, putative
LmjF.09.0880:mRNA	histone h1-like protein
LmjF.30.0670:mRNA	40S ribosomal protein S30, putative
LmjF.30.0680:mRNA	40S ribosomal protein S30, putative
LmjF.30.0690:mRNA	40S ribosomal protein S30, putative
LmjF.27.1580:mRNA	amino acid transporter, putative
LmjF.31.0320:mRNA	amino acid transporter, putative
LmjF.36.4480:mRNA	amino acid transporter, putative
